# Safety of bariatric surgery in patients with previous acute coronary events or heart failure: nationwide cohort study

**DOI:** 10.1093/bjsopen/zrac083

**Published:** 2022-06-09

**Authors:** Erik Stenberg, Yang Cao, Tomas Jernberg, Erik Näslund

**Affiliations:** Department of Surgery, Faculty of Medicine and Health, Örebro University, Örebro, Sweden; Clinical Epidemiology and Biostatistics, School of Medical Sciences, Örebro University, Örebro, Sweden; Division of Cardiovascular Medicine, Department of Clinical Sciences, Danderyd Hospital, Karolinska Institutet, Stockholm, Sweden; Division of Surgery, Department of Clinical Sciences, Danderyd Hospital, Karolinska Institutet, Stockholm, Sweden

## Abstract

**Background:**

Metabolic (bariatric) surgery for patients with severe obesity and pre-existing heart disease has been reported to reduce the risk for cardiovascular events and mortality; however, concerns of short- and mid-term complications may limit the utility of metabolic surgery for these patients.

**Method:**

This was an observational, nationwide, matched study, including all adult patients operated with a primary gastric bypass or sleeve gastrectomy procedure in Sweden from January 2011 until October 2020. Patients with or without previous acute coronary syndrome or heart failure were matched 1:5 using propensity scores. The primary outcome was serious postoperative complications, and secondary outcomes were the occurrence of any short-term complications, mid-term complications, weight loss, and health-related quality of life estimates after surgery

**Results:**

Of patients who underwent metabolic surgery, 1165 patients with previous acute coronary syndrome or heart failure and 5825 without diagnosed heart disease were included in matched analyses. No difference was seen between the groups at risk for serious postoperative complications within 30 days of surgery (OR 1.33, 95 per cent c.i. 0.95 to 1.86, *P* = 0.094), whereas heart disease was associated with an increased risk for cardiovascular complications (incidence 1.1 per cent *versus* 0.2 per cent, *P* < 0.001). No differences in overall mid-term complications, weight loss, or improvement of health-related quality of life were seen. Pre-existing heart disease was associated with an increased risk for bowel obstruction and strictures (OR 1.89, 95 per cent c.i. 1.20 to 2.99, *P* = 0.006).

**Conclusion:**

Patients with severe obesity and heart disease undergoing metabolic surgery have an increased risk of postoperative cardiovascular complications compared with patients with severe obesity without heart disease. A careful preoperative cardiovascular work-up is needed but patients with severe obesity and heart disease should not be excluded from undergoing metabolic surgery.

## Teaser

In this nationwide, matched study of 1165 patients with previous acute coronary syndrome or heart failure and 5825 controls without heart disease who underwent sleeve gastrectomy or Roux-en-Y gastric bypass, no difference was seen in the risk of serious postoperative complications, weight loss, or health-related quality of life. An increased risk for cardiovascular complications was seen.

## Introduction

There is a clear association between severe obesity and type 2 diabetes (T2D) and the associated insulin resistance, dyslipidaemia, and hypertension. These co-morbidities also increase the risk for cardiovascular disease. The evidence for treating T2D in patients with severe obesity with metabolic (bariatric) surgery is strong following several randomized clinical trials (RCTs)^1,2^. Over the past decade there is also increasing evidence from observational studies that metabolic surgery significantly reduces the risk for cardiovascular disease such as myocardial infarction, stroke, and cardiovascular death^3^. Metabolic surgery was associated with a reduced rate of all-cause and cardiovascular death in a recent meta-analysis of 18 observational studies^4^. There is currently one RCT that has shown a superior effect of metabolic surgery on hypertension compared with conventional treatment^5^.

While metabolic surgery has a role in primary prevention, it has been proposed that metabolic surgery has also a role in secondary prevention of cardiovascular disease. Metabolic surgery is associated with a lower risk of major adverse cardiovascular events (MACEs) in patients with severe obesity and hypertension^6^, a previous myocardial infarction^7^, or ischaemic heart disease or heart failure^8^, and the risk of MACE is reduced by half in patients that go into remission of their hypertension compared with those who do not^9^. One of the reasons why cardiologists and surgeons might be reluctant to suggest metabolic surgery to a patient with pre-existing cardiovascular disease is a paucity in data regarding short- and mid-term postoperative complications^10,11^.

The aim of the present study was to assess both short- and mid-term complications in patients with severe obesity and pre-existing heart disease who undergo metabolic surgery.

## Methods

This observational, matched cohort study was based on data from the Scandinavian Obesity Surgery Registry (SOReg), a national research and quality register that started in 2007 covering virtually all bariatric surgical procedures in Sweden (52 reporting centres during the study interval). The registry is continuously validated and registrations have so far been shown to have very high validity^12^. By using the national identification numbers (unique to all Swedish residents), the SOReg database was linked to the National Patient Register (NPR) for inpatient and outpatient care as well as the Swedish Prescribed Drugs Register, the Swedish Cause of Death Registry, Total Population Registry, and the Education Register from Statistics Sweden for individual information on level of highest education. The NPR is a nationwide register that attained national coverage in 1987 covering all hospital admissions in public healthcare, while the outpatient component started in 2001 and covers about 95 per cent of outpatient visits in specialized healthcare^13^. The Swedish Prescribed Drugs Register was established in 2005 and includes all dispensed drugs classified according to the WHO Anatomical Therapeutic Chemical (ATC) classification system^14^.

### Inclusion and exclusion

While there were no mandatory national eligibility criteria for metabolic surgery in Sweden during the study interval, most regions in Sweden considered a slightly more liberal approach compared with the National Institutes of Health criteria from 1991^15^, with BMI of 35 kg/m^2^ or more with or without co-morbidity as an eligibility criterion.

All patients aged at least 18 years who had a primary sleeve gastrectomy or gastric bypass from 1 January 2011 until 8 October 2020 were included in the study. Heart disease was specified as previous history of an acute coronary syndrome (ACS), heart failure, or cardiomyopathy, defined as hospital admission for unstable angina pectoris (ICD-10, I20.0), myocardial infarction (ICD-10, I21–22), heart failure (ICD-10, I50) or cardiomyopathy (ICD-10, I42) before surgery, or a diagnosis of heart failure or cardiomyopathy in the NPR for outpatient care if dispensed loop diuretics (ATC-code, C03C), beta-blockers (ATC-code, C07A), ACE-inhibitor or angiotensin-II inhibitor (ATC-code, C09A, C09B, or C09C) within 12 months before surgery.

### Matching procedure

Patients with a preoperative episode of ACS or heart failure were matched 1:5 with controls who underwent metabolic surgery without a preoperative diagnosis of ACS or heart failure. The matching was conducted as a two-stage procedure with a first exact match on surgical method (bypass or sleeve), followed by a propensity score match (nearest function without limit for calliper), including age, sex, preoperative BMI, surgical centre, year of surgery, hypertension, diabetes, sleep apnoea, dyslipidaemia, depression, and level of education.

### Definitions

Co-morbidity was defined as an obesity-related condition (specified as diabetes, dyslipidaemia, hypertension, or sleep apnoea), or depression requiring active pharmacological or continuous positive-airway pressure treatment.

History of smoking was based on self-reporting at baseline.

Level of education was divided into three categories based on the highest completed education at the time of surgery: primary education (9 years of schooling or fewer), secondary education (completed 10–12 years of schooling), or higher education (completed college or university degree).

### Procedures

The surgical technique for the laparoscopic gastric bypass procedure was highly standardized in Sweden during the study interval and was an antecolic, antegastric Roux-en-Y gastric bypass (RYGB) with a small gastric pouch (less than 25 ml), an alimentary limb of 100 cm, and a biliopancreatic limb of 50 cm. The sleeve gastrectomy (SG) was less standardized, but most operations were routinely performed using a 32–36 Fr Bougie, starting the resection 5 cm or less from the pylorus, ending the resection 1 cm lateral to the angle of His.

### Endpoints

The main outcome was serious postoperative complication within 30 days of surgery. A serious postoperative complication was defined as a complication graded 3b or above on the Clavien–Dindo scale (a complication requiring an intervention under general anaesthesia, single or multiple organ failure, or death)^16^. Data on mortality were based on combined data from the total population registry (reporting complete coverage of mortality), and the SOReg, NPR, and Cause of Death Registry for cause of death. Secondary outcomes were the occurrence of any short-term postoperative complication within 30 days of surgery (defined as a specific complication requiring prolonged duration of hospital stay, readmission, or intervention, thus deviating from a normal postoperative course), mid-term complications (30 day – 2 year follow-up, defined as treatment, or readmission for anaemia, malnutrition, marginal ulcer, bowel obstruction/stricture, or leak/gastric or intestinal perforation), postoperative weight loss measured as percentage total weight loss (TWL), excess BMI loss (EBMIL), and BMI loss in accordance with current recommendations^17^, and health-related quality of life (HRQoL). HRQoL was estimated with the physical and mental components summary score using RAND-36/^®^ (SF-36/RAND^®^) scale (RAND Corp, USA)^18^, as well as the disease-specific obesity problems (OP) scale^19^. Short-term complications were also compared across procedures (RYGB and SG) and stratified by ACS without heart failure, and heart failure alone.

### Statistics

Continuous variables assuming a normal distribution are presented as the mean(s.d.) or as the median with interquartile range (i.q.r.) when not assuming a normal distribution. Categorical variables are presented as numbers (*n*) and proportions (per cent). Differences in proportions were evaluated with conditional logistic regression or the Fisher’s exact test. Associations of heart disease with binary outcomes were assessed using a conditional logistic regression model, with continuous outcome variables using a linear mixed-effects model, and with HRQoL outcomes using a linear quantile mixed-effects model with fixed variables and random intercepts for each matched group. After matching, the balance between cases and controls was evaluated by calculating the standardized difference, measuring difference in units of the pooled s.d. Based on this balance evaluation, all the analyses were conditional to matching and adjusted for smoking. Interaction between heart disease and smoking, if present, was also tested in the mixed-effects models. The OR, mean difference (MeD), and median difference (MdD) are presented as measures of association. Due to multiple calculations of secondary endpoints, the Bonferroni–Holm method was used to adjust for multiple comparisons^20^. Missing data were handled by listwise deletion.

SPSS^®^ version 25 (IBM, Armonk, New York, USA) and Stata version 17.0 (StataCorp, College Station, Texas, USA) were used for statistical analyses.

### Ethics

The study was approved by the Swedish Ethical Review Authority (ref. no. 2020-03005) and conducted in accordance with the standards of the 1964 Helsinki Declaration and its later amendments.

## Results

During the study interval, 56 999 patients were identified in the Scandinavian Obesity Surgery Registry. A total of 1165 patients met the criteria with primary SG/RYGB and previous history of ACS, or heart failure. The matching procedure resulted in two groups without any relevant differences in patient characteristics (*Table 1*).

**Table 1:** Baseline characteristics and surgical procedure after propensity score matching

	Heart disease group	Control group	Standardized difference
**Patients**	1165	5825	
**Age (years), mean (s.d.)**	53.0(8.1)	52.9(7.9)	0.012
**BMI (kg/m^2^), mean (s.d.)**	42.0(5.6)	41.9(5.5)	0.018
**Sex**			
Women	505 (43.3)	2747 (47.2)	−0.078
Men	660 (56.7)	3078 (52.8)	0.078
**Metabolic co-morbidities**			
Diabetes	508 (43.6)	2493 (42.8)	0.016
Dyslipidaemia	643 (55.2)	3012 (51.7)	0.070
Hypertension	929 (79.7)	4777 (82.0)	−0.058
Sleep apnoea	352 (30.2)	1625 (27.9)	0.051
**Other co-morbidities**			
Depression	190 (16.3)	950 (16.3)	0
**Smoking** *			
Active smoking	98 (9.3)	485 (9.1)	0.007
Previous smoking	296 (28.0)	1280 (24.0)	0.091
None	662 (62.7)	3570 (66.9)	−0.088
**Education** †			
Primary education (9 years)	270 (23.2)	1366 (23.5)	−0.007
Secondary education (10–12 years)	699 (60.1)	3325 (57.1)	0.061
Higher education	194 (16.7)	1134 (19.5)	−0.073
**Operation**			
Gastric bypass	974 (83.6)	4870 (83.6)	0
Sleeve gastrectomy	191 (16.4)	955 (16.4)	0

Numbers are *n* (%) unless otherwise indicated.

* Missing data for 109 (9.4 per cent) patients in the heart disease group, and 490 (8.4 per cent) in the control group.

†Missing data on highest level of education for one patient in the heart disease group (0.0 per cent) and one patient in the control group (0.0 per cent).

Information on the operation and intraoperative complications were available for all patients. Follow-up for postoperative complications at day 30 was available for 1131 patients in the heart disease group (97.1 per cent), and 5687 in the control group (97.6 per cent). The follow-up rate for weight loss was 88.0 per cent (*n* = 984 of 1118) in the heart disease group and 89.2 per cent (*n* = 5022 of 5630) in the control group at one year, and 64.9 per cent (*n* = 657 of 1013) in the heart disease group and 69.2 per cent (*n* = 3581 of 5176) in the control group at two years after surgery. HRQoL is reported with some delay and only at some of the included surgical centres (44 centres reported HRQoL data, but not all centres reported all years). Therefore, information on HRQoL was available at baseline for 788 patients in the heart disease group at baseline and 3970 patients in the control group.

### Short-term safety outcomes

Mean operating time was 72.0(35.8) min in the heart disease group *versus* 70.2(33.5) min in the control group (*P* = 0.185). No difference in the risk for intraoperative complications was seen (*n* = 33 (2.8 per cent) in the heart disease group *versus n* = 186 (3.2 per cent) in the control group; OR 0.89, 95 per cent c.i. 0.61 to 1.30, *P* = 0.543). Median duration of hospital stay was 1 (i.q.r. 1–2) day in the heart disease group *versus* 1 (i.q.r. 1–2) day in the control group (*P* = 0.993).

No difference in the risk for overall complications, nor serious postoperative complications was seen. A higher risk for cardiovascular complications during the first 30 days was seen in the heart disease group compared with the control group (1.1 per cent *versus* 0.2 per cent, *P* < 0.001). Death within 90 days of surgery was also more common in the group with previous heart disease (*n* = 8; 0.7 per cent), compared with *n* = 2 (0.03 per cent), in the control group (*P* < 0.001). All patients who died in the group with previous heart disease underwent a RYGB, whereas both patients in the control group underwent an SG. The cause of death for patients with heart disease was a cardiovascular event for five patients, septicaemia in two, and unknown for one patient. The causes of death for the controls were an acute cerebrovascular event for one patient and pulmonary embolism for one patient. No other significant differences were seen between the two groups in short-term postoperative safety outcomes (*Table 2*).

**Table 2:** Surgical outcomes

	Heart disease group	Control group	Effect size OR or MeD* (95 per cent c.i.)	*P*
**Intraoperative complication** ‡	33 (2.8)	186 (3.2)	0.89 (0.61 to 1.30)	0.543
**Postoperative complication** ‡	106 (9.4)	464 (8.2)	1.14 (0.92 to 1.43)	0.231
Leak/deep intraabdominal infection‡	23 (2.0)	104 (1.8)	1.12 (0.71 to 1.78)	0.629
Bleeding‡	29 (2.6)	118 (2.1)	1.20 (0.79 to 1.82)	0.395
Wound complications‡	17 (1.5)	71 (1.2)	1.15 (0.67 to 1.97)	0.620
Bowel obstruction/stricture/ileus‡	16 (1.4)	58 (1.0)	1.34 (0.76 to 2.36)	0.310
Marginal ulcer‡	4 (0.4)	24 (0.4)	0.85 (0.29 to 2.48)	0.767
Cardiovascular complication‡	12 (1.1)	12 (0.2)	6.56 (2.67 to 16.15)	<0.001†
Pulmonary complication‡	11 (1.0)	35 (0.6)	1.71 (0.86 to 3.43)	0.128
Urinary tract infection‡	10 (0.9)	19 (0.3)	2.49 (1.14 to 5.45)	0.242
Venous thrombosis‡	1 (0.1)	8 (0.1)	0.69 (0.08 to 5.96)	0.735
Pain‡	7 (0.6)	41 (0.7)	0.71 (0.30 to 1.67)	0.430
Malnutrition/dehydration‡	7 (0.6)	27 (0.5)	1.38 (0.58 to 3.30)	0.464
Other complication‡	9 (0.8)	54 (0.9)	0.84 (0.41 to 1.70)	0.628
**Serious postoperative complication** ‡ §	46 (4.1)	179 (3.1)	1.33 (0.95 to 1.86)	0.094
**90-day mortality**	8 (0.7)	2 (0.0)	34.98 (4.29 to 285.42)	<0.001†
**Postoperative weight loss at 1 year**				
EBMIL¶, mean(s.d.)	74.6(23.5)	73.6(22.5)	1.00 (−0.60 to 2.40)	0.238
TWL¶, mean(s.d.)	28.7(8.1)	28.3(7.8)	−0.41 (−0.92 to 0.11)	0.119
BMI loss¶, mean(s.d.)	12.1(4.1)	11.9(4.0)	0.18 (−0.08 to 0.44)	0.180
**Postoperative weight loss at 2 years**				
Per cent EBMIL¶, mean(s.d.)	74.3(24.8)	73.9(24.3)	0.32 (−1.67 to 2.31)	0.752
Per cent TWL¶, mean(s.d.)	28.7(9.1)	28.4(9.0)	0.26 (−1.00 to 0.47)	0.479
BMI loss¶, mean(s.d.)	12.1(4.5)	12.0(4.5)	−0.13 (−0.50 to 0.25)	0.509

Numbers are *n* (%), unless otherwise indicated. EBMIL, excess BMI loss; TWL, total weight loss; MeD, mean difference.

* OR for categorical, MeD for continuous variables.

†Significant difference (*P* < 0.05) after corrections for multiple calculations using the Bonferroni–Holm method.

‡Conditional logistic regression model, adjusted for smoking.

§Serious postoperative complications include any complication occurring with 30 days after surgery, requiring an intervention under general anaesthesia, or resulting in single or multiple organ failure, or death.

¶Linear mixed-effects model, adjusted for smoking

When stratified by surgical approach, no difference was seen in overall occurrence of postoperative complications (9.0 per cent in the heart disease group compared with 8.7 per cent in the control group, OR 1.04, 95 per cent c.i. 0.81 to 1.32, *P* = 0.770), nor serious postoperative complications (3.9 per cent *versus* 3.3 per cent, OR 1.21, 95 per cent c.i. 0.83 to 1.75, *P* = 0.320) for RYGB. An increased risk for cardiovascular complications was seen for patients with heart disease after RYGB (1.0 per cent *versus* 0.2 per cent, OR 6.84, 95 per cent c.i. 2.47 to 18.90, *P* < 0.001).

An increased risk for any postoperative complication (11.2 per cent *versus* 5.5 per cent, OR 2.11, 95 per cent c.i. 1.21 to 3.67, *P* = 0.008) as well as serious postoperative complication (5.1 per cent *versus* 2.2 per cent, OR 2.30, 95 per cent c.i. 1.01 to 5.21, *P* = 0.046) was seen after SG in patients with heart disease compared with the control group. No statistically significant difference was noted for any of the specified complications for SG (*Tables S1 and S2*).

A lower risk for an intraoperative complication (1.8 per cent *versus* 3.6 per cent, OR 0.45, 95 per cent c.i. 0.25 to 0.92, *P* = 0.028) and a higher weight loss (as measured by EBMIL) both after the 1- and 2-year follow-up was seen in patients with ACS without heart failure compared with controls (*Table S3*). Patients with heart failure alone had an increased risk for a cardiovascular complication (1.2 per cent *versus* 0.2 per cent, OR 7.90, 95 per cent c.i. 2.20 to 28.37, *P* = 0.024) and a higher BMI loss after the 1-year follow-up compared with controls (*Table S4*). Similar patterns in complications were seen when data were stratified according to BMI (*Tables S5 and S6*).

### Mid-term safety outcomes

No difference in overall complication rate from day 30 until 2 years after surgery was seen between the groups; however, with regard to specific complications, bowel obstruction or strictures occurred more often in the heart disease group (*Table 3*).

**Table 3:** Mid-term (between 30 days and 2 years) complications

	Heart disease	Control group	OR (95 per cent c.i.)	*P*
**Any complication**	61 (8.9)	249 (6.6)	0.33 (0.08 to 1.32)*	0.118*
**Specific complication**				
Anaemia	17 (2.5)	66 (1.8)	1.43 (0.78 to 2.63)†	0.250†
Malnutrition	6 (0.9)	18 (0.5)	2.14 (0.72 to 6.38)†	0.076†
Marginal ulcer	24 (3.5)	97 (2.6)	1.33 (0.80 to 2.22)†	0.270†
Bowel obstruction/stricture‡	31 (4.5)	99 (2.7)	1.89 (1.20 to 2.99)†	0.006†
Leak/ perforation	4 (0.6)	11 (0.3)	2.12 (0.47 to 9.55)†	0.327†

Numbers are *n* (%) unless otherwise indicated.

* Conditional logistic regression, adjusted for smoking, and interaction between heart disease and smoking.

†Conditional logistic regression, adjusted for smoking; interaction term was not statistically significant and excluded in the model.

‡The mesenteric defect beneath the jejunojejunostomy was closed for 87 per cent in the heart disease group and 86 per cent in the control group. Petersen’s space was closed during 84 per cent of the operations in both groups.

### Weight outcome

Mean(s.d.) BMI loss at 1 year after surgery for all patients in the study was 12.0(3.99) kg/m^2^, mean EBMIL 73.7(22.63) per cent and TWL 28.4(7.87) per cent with no differences between the heart disease group and the control group (*Table 2*). Mean(s.d.) BMI loss at 2 years was 12.0(4.54) kg/m^2^, mean(s.d.) EBMIL was 74.0(24.41) per cent and TWL was 28.4(9.02) per cent with no differences between the heart disease group and the control group (*Table 2*).

### HRQoL

Patients with heart disease reported a lower physical quality of life before surgery compared with the control group. All aspects of HRQoL, as estimated with the summary scores from SF-36 and OP, improved for both groups, and no difference was seen between the groups either at 1 or 2 years after surgery (*Table 4*).

**Table 4:** Health-related quality of life

	Heart disease		Control group		Median difference (95 per cent c.i.)	*P* ^†^
	*n* *	Median (i.q.r.)	*n* *	Median (i.q.r.)		
**OP score, baseline**	788	62.5 (33.3–79.2)	3970	62.5 (33.3–79.2)	−0.34 (−2.34 to 1.66)	0.737
**OP score, 1 year**	598	8.3 (0.0–25.0)	3211	8.3 (0.0–25.0)	−0.63 (−3.74 to 2.49)	0.693
**OP score, 2 years**	372	8.3 (0.0–29.2)	2200	8.3 (0.0–29.2)	1.11 (−7.11 to 9.33)	0.792
**SF-36/RAND**						
**Mental component, baseline**	773	50.4 (39.1–56.6)	3870	50.1 (38.7–56.2)	0.01 (−0.85 to 0.87)	0.986
**Mental component, 1 year**	585	54.0 (45.2–57.5)	3148	54.3 (46.6–57.3)	−0.21 (−3.61 to 3.19)	0.904
**Mental component, 2 years**	358	52.8 (38.3–57.0)	2164	53.4 (44.9–56.9)	−1.45 (−4.28 to 1.38)	0.315
**Physical component, baseline**	773	34.2 (26.0–43.7)	3872	35.7 (27.1–44.0)	−1.16 (−1.97 to −0.36)	0.004‡
**Physical component, 1 year**	583	50.4 (41.7–55.5)	3144	52.0 (44.6–56.2)	−0.70 (−3.31 to 1.91)	0.600
**Physical component, 2 years**	358	50.0 (41.32–55.5)	2160	51.8 (43.4–56.1)	−0.75 (−2.91 to 1.41)	0.498

OP, obesity problems scale; SF-36^®^/RAND, Short Form 36^®^; i.q.r., interquartile range.

* Number of correctly filled out evaluations.

†Linear quantile mixed-effects model, adjusted for smoking, and baseline values.

‡Significant difference (*P* < 0.05) after corrections for multiple calculations using the Bonferroni–Holm method.

## Discussion

This study demonstrates that metabolic surgery can be performed safely in patients with previous heart disease (acute cardiovascular event, heart failure, or cardiomyopathy). The overall risk for early (less than 30 days) and late (30 days or more) complications was similar for patients with cardiovascular disease and the matched group that did not have cardiovascular disease; however, there was an increased risk for early cardiovascular complications, a higher 90-day mortality rate, and an increased risk for bowel obstruction or strictures in patients with previous heart disease. There was an increased risk for any early complications after SG compared with the control group; however, cardiovascular complications were only increased after RYGB in patients with previous heart disease. There was no difference in HRQoL after metabolic surgery in patients with or without previous heart disease. There was no difference in weight loss at 1 or 2 years of follow-up.

Although the number of patients is low, in equivalence with a recent study from North America^11^, there was a 4.5-fold relative risk for cardiovascular complications in the group with previous heart disease; however, this needs to be put into the context of a significant reduction in both MACE and mortality seen after metabolic surgery in patients with previous cardiovascular disease^7, 8^. The overall 90-day mortality seen in this study (0.1 per cent) is comparable to that reported in the literature (0.03–0.2 per cent)^21^. For those without previous heart disease the 90-day mortality rate was very low and for those with previous heart disease was slightly higher than rates reported in the literature. This elevated risk was driven by cardiovascular death. Although patients with previous heart disease had an increased risk for early postoperative mortality, the overall risk for mortality remains lower up to 8 years after surgery when compared with patients with heart disease who did not undergo metabolic surgery^7^.

Bowel obstruction or strictures were higher in the group with previous heart disease. The reason for this is not clear. There was no difference in mesenteric defects closure between the two groups. It is possible that patients in the group with previous heart disease were more likely to use medication that may increase the risk for ulcers and strictures. Also, patients with heart failure are more prone to have portal congestion and intestinal oedema^22^. The two groups were well matched with regard to preoperative co-morbidity other than heart disease, which makes it unlikely that differences in postoperative co-morbidity might influence the risk for late complications. Due to a small number of events, we could not stratify mid-term complications according to surgical procedure. However, a meta-analysis of published RCTs comparing late complications after RYGB and SG found no significant difference between the procedures^23^.

Current data regarding the role of metabolic surgery in the secondary prevention of cardiovascular disease are based on observational studies. Thus, patient recommendations need to be given with caution and no firm guidelines exist. Despite the strengths of the large nationwide study population and the use of high-quality data from several sources, this study has several limitations. First, this is an observational study, residual confounding factors may exist, and causality cannot be shown. As with all registry studies, coding errors may exist. On the other hand, this study was based on large nationwide registers with known high validity and degree of completeness. Second, the outcomes may differ depending on surgical method, BMI, sex, and severity of disease. When stratifying the results, on surgical method, type of heart disease, BMI, or sex, no major differences that would have an impact on the conclusions were seen; however, no differentiation of the severity of T2D was included in the study. While this was not an aim of the present study, the outcomes of metabolic surgery for patients with severe heart disease and T2D of different severity should preferably be the focus of future studies.

Although a careful preoperative cardiovascular work-up is needed, patients with severe obesity and heart disease should not be excluded from undergoing metabolic surgery considering the recently demonstrated positive effects of metabolic surgery in secondary cardiovascular prevention.

## Supplementary Material

zrac083_Supplementary_Data

## Data Availability

Swedish data protection laws do not allow for the sharing of patient data.
