# Supplementary material for: Safety of bariatric surgery in patients with previous acute coronary events or heart failure: nationwide cohort study
Source: BJS Open. 2022 Jun 9;6(3):zrac083. doi: 10.1093/bjsopen/zrac083 (PMC9177412; doi:10.1093/bjsopen/zrac083)
Supplement: zrac083_Supplementary_Data [file zrac083_supplementary_data.docx]

| **Table S1. Surgical outcomes after gastric bypass** | | | |  |
| --- | --- | --- | --- | --- |
|  | Heart disease | Control group | *Effect size (95% CI)* | *P* |
| Number of individuals | 974 | 4870 |  |  |
| Length of hospital stay, days median (IQR)^1^ | 1 (1-2) | 1 (1-2) | MdD=0.07 (-0.98 – 1.11) | 0.897 |
| Intraoperative complication^2^ | 31 (3.2%) | 164 (3.4%) | OR=0.95 (0.64 – 1.41) | 0.799 |
| Postoperative complication^2^ | 86 (9.0%) | 413 (8.7%) | OR=1.04 (0.81 – 1.32) | 0.770 |
| Leak/deep intraabdominal infection^2^ | 19 (2.0%) | 96 (2.0%) | OR=0.99 (0.60 – 1.64) | 0.969 |
| Bleeding^2^ | 24 (2.5%) | 102 (2.1%) | OR=1.15 (0.72 – 1.82) | 0.551 |
| Wound complications^2^ | 13 (1.4%) | 62 (1.3%) | OR=1.00 (0.54 – 1.86) | 0.992 |
| Bowel obstruction/stricture/ileus^2^ | 15 (1.6%) | 55 (1.1%) | OR=1.32 (0.74 – 2.36) | 0.354 |
| Marginal ulcer^2^ | 4 (0.4%) | 23 (0.5%) | OR=0.90 (0.31 – 2.63) | 0.844 |
| Cardiovascular complication^2^ | 10 (1.0%) | 10 (0.2%) | OR=6.84 (2.47 – 18.90) | <0.001* |
| Pulmonary complication^2^ | 10 (1.0%) | 30 (0.6%) | OR=1.79 (0.117 – 3.72) | 0.117 |
| Urinary tract infection^2^ | 6 (0.6%) | 15 (0.3%) | OR=1.95 (0.75 – 5.10) | 0.173 |
| Venous thrombosis^2^ | 0 (0.0%) | 5 (0.1%) | NA |  |
| Pain^2^ | 5 (0.5%) | 37 (0.8%) | OR=0.58 (0.21 – 1.59) | 0.287 |
| Malnutrition/dehydration^2^ | 5 (0.5%) | 23 (0.5%) | OR=1.24 (0.46 – 3.34) | 0.668 |
| Other complication^2^ | 8 (0.8%) | 49 (1.0%) | OR=0.82 (0.38 -1.73) | 0.602 |
| Serious postoperative complication^2^ | 37 (3.9%) | 159 (3.3%) | OR=1.21 (0.83 – 1.75) | 0.320 |
| Postoperative weight-loss at 1 year |  |  |  |  |
| %EBMIL^3^, mean ± SD | 76.1 ± 23.2 | 75.3 ± 21.8 | MeD=0.70 (-0.89 – 2.30) | 0.390 |
| %TWL^3^, mean ± SD | 29.4 ± 7.7 | 29.1 ± 7.5 | MeD=-0.31 (-0.85 – 0.23) | 0.261 |
| BMI-loss^3^, mean ± SD | 12.4 ± 3.9 | 12.3 ± 3.9 | MeD=-0.15 (-0.43 – 0.13) | 0.298 |
| Postoperative weight-loss at 2 years |  |  |  |  |
| %EBMIL^3^, mean ± SD | 75.9± 24.4 | 75.7 ± 23.6 | MeD=0.03 (-0.21 – 0.21) | 0.980 |
| %TWL^3^, mean ± SD | 29.4 ± 8.9 | 29.2 ± 8.7 | MeD=-0.13 (-0.91 – 0.63) | 0.726 |
| BMI-loss^3^, mean ± SD | 12.4 ± 4.4 | 12.4 ± 4.5 | MeD=-0.07 (-0.46 – 0.33) | 0.737 |
| Numbers are n (%) unless otherwise stated  EBMIL = Excess BMI-loss; TWL = Total Weight Loss  1 Linear quantile mixed-effects model, adjusted for smoking; MdD, median difference.  2 Conditional logistic regression model, adjusted for smoking; OR, odds ratio.  3 Linear mixed-effects model, adjusted for smoking; MeD, mean difference.  * Corrected value for multiple calculations using the Bonferroni-Holm method | | | |  |

| **Table S2 Surgical outcomes after sleeve gastrectomy** | | | |  |
| --- | --- | --- | --- | --- |
|  | Heart disease | Control group | *Effect size (95% CI)* | *P* |
| Number of individuals | 191 | 955 |  |  |
| Length of hospital stay, days median (IQR)^1^ | 1 (1-2) | 1 (1-2) | MdD=0.10 (-1.75 – 1.94) | 0.918 |
| Intraoperative complication^2^ | 2 (1.0%) | 22 (2.3%) | OR=0.46 (0.11- 2.00) | 0.302 |
| Postoperative complication^2^ | 20 (11.2%) | 51 (5.5%) | OR=2.11 (1.21 – 3.67) | 0.008 |
| Leak/deep intraabdominal infection^2^ | 4 (2.2%) | 8 (0.9%) | OR=3.05 (0.84 – 11.07) | 0.090 |
| Bleeding^2^ | 5 (2.8%) | 16 (1.7%) | OR=1.27 (0.42 – 3.81) | 0.669 |
| Wound complications^2^ | 4 (2.2%) | 9 (1.0%) | OR=2.89 (0.79 – 10.64) | 0.110 |
| Bowel obstruction/stricture/ileus^2^ | 1 (0.6%) | 3 (0.3%) | OR=5.09 (0.28 – 94.28) | 0.274 |
| Marginal ulcer^2^ | 0 (0.0%) | 1 (0.1%) | NA |  |
| Cardiovascular complication^2^ | 2 (1.1%) | 2 (0.2%) | OR=2.86 (0.43 – 19.07) | 0.277 |
| Pulmonary complication^2^ | 1 (0.6%) | 5 (0.5%) | OR=0.77 (0.05 – 10.97) | 0.844 |
| Urinary tract infection^2^ | 4 (2.2%) | 4 (0.4%) | OR=4.82 (0.87 – 26.81) | 0.073 |
| Venous thrombosis^2^ | 1 (0.6%) | 3 (0.3%) | OR=1.36 (0.23 – 7.92) | 0.731 |
| Pain^2^ | 2 (1.1%) | 4 (0.4%) | OR=2.13 (0.33 – 13.95) | 0.430 |
| Malnutrition/dehydration^2^ | 2 (1.1%) | 4 (0.4%) | OR=2.50 (0.46 – 13.65) | 0.290 |
| Other complication^2^ | 1 (0.6%) | 5 (0.5%) | OR=0.81 (0.09 – 7.07) | 0.851 |
| Serious postoperative complication^2^ | 9 (5.1%) | 20 (2.2%) | OR=2.30 (1.01 – 5.21) | 0.046 |
| Postoperative weight-loss at 1 year |  |  |  |  |
| %EBMIL^3^, mean ± SD | 64.8 ± 23.5 | 63.0 ± 23.6 | MeD=2.02 (-2.23 – 6.28) | 0.352 |
| %TWL^3^, mean ± SD | 24.4 ± 9.1 | 23.6 ± 8.2 | MeD=-0.87 (-2.39 – 0.65) | 0.263 |
| BMI-loss^3^, mean ± SD | 10.1 ± 4.3 | 9.8 ± 4.0 | MeD=-0.29 (-1.03 – 0.56) | 0.449 |
| Postoperative weight-loss at 2 years |  |  |  |  |
| %EBMIL^3^, mean ± SD | 63.8 ± 25.6 | 61.7 ± 25.2 | MeD=2.18 (-3.78 – 8.14) | 0.473 |
| %TWL^3^, mean ± SD | 23.9 ± 9.6 | 22.9 ± 9.0 | MeD=-0.94 (-3.09 – 1.21) | 0.394 |
| BMI-loss^3^, mean ± SD | 9.9 ± 4.5 | 9.5 ± 4.2 | MeD=-0.40 (-1.41 – 0.61) | 0.441 |
| Numbers are n (%) unless otherwise stated  EBMIL = Excess BMI-loss; TWL = Total Weight Loss  1 Linear quantile mixed-effects model, adjusted for smoking; MdD, median difference.  2 Conditional logistic regression model, adjusted for smoking; OR, odds ratio.  3 Linear mixed-effects model, adjusted for smoking; MeD, mean difference. | | | |  |

| **Table S3. Surgical outcomes for patients with Acute Coronary Syndrome without heart failure** | | | |  |
| --- | --- | --- | --- | --- |
|  | ACS | Control group | *Effect size (95% CI)* | *P* |
| Number of individuals | 573 | 2865 |  |  |
| Length of hospital stay (IQR)^1^ | 1 (1-2) | 1 (1-2) | MdD=-0.02 (-0.45 – 0.49) | 0.932 |
| Intraoperative complication^2^ | 10 (1.7%) | 102 (3.6%) | OR=0.45 (0.25 – 0.92) | 0.028 |
| Postoperative complication^2^ | 52 (9.4%) | 229 (8.2%) | OR=1.13 (0.82 – 1.56) | 0.445 |
| Leak/deep intraabdominal infection^2^ | 9 (1.6%) | 51 (1.8%) | OR=0.88 (0.42 – 1.85) | 0.734 |
| Bleeding^2^ | 16 (2.9%) | 55 (2.0%) | OR=1.40 (0.78 – 2.50) | 0.263 |
| Wound complications^2^ | 7 (1.3%) | 39 (1.4%) | OR=0.86 (0.38 – 1.97) | 0.730 |
| Bowel obstruction/stricture/ileus^2^ | 10 (1.8%) | 33 (1.2%) | OR=1.58 (0.76 – 3.26) | 0.218 |
| Marginal ulcer^2^ | 0 (0.0%) | 8 (0.3%) | NA |  |
| Cardiovascular complication^2^ | 5 (0.9%) | 5 (0.2%) | OR=10.21 (1.70 – 60.99) | 0.132* |
| Pulmonary complication^2^ | 5 (0.9%) | 16 (0.6%) | OR=1.56 (0.55 – 4.45) | 0.405 |
| Urinary tract infection^2^ | 5 (0.9%) | 9 (0.3%) | OR=2.99 (0.91 – 9.86) | 0.071 |
| Venous thrombosis^2^ | 1 (0.2%) | 2 (0.1%) | OR=3.64 (0.25 – 52.55) | 0.343 |
| Pain^2^ | 3 (0.5%) | 16 (0.6%) | OR=0.82 (0.21 – 3.16) | 0.774 |
| Malnutrition/dehydration^2^ | 3 (0.5%) | 14 (0.5%) | OR=1.07 (0.28 – 4.05) | 0.923 |
| Other complication^2^ | 7 (1.3%) | 32 (1.1%) | OR=1.17 (0.51 -2.69) | 0.704 |
| Serious postoperative complication^2^ | 22 (4.0%) | 90 (3.2%) | OR=1.20 (0.70 – 2.06) | 0.501 |
| Postoperative weight-loss at 1 year |  |  |  |  |
| %EBMIL^3^, mean ± SD | 77.9 ± 24.6 | 73.8 ± 22.5 | MeD= 3.97 (1.78 – 6.17) | <0.001 |
| %TWL^3^, mean ± SD | 28.7 ± 8.1 | 28.2 ± 7.8 | MeD= 0.42 (-0.32 – 1.16) | 0.267 |
| BMI-loss^3^, mean ± SD | 11.7 ± 3.8 | 11.9 ± 4.0 | MeD=-0.17 (-0.55 – 0.20) | 0.365 |
| Postoperative weight-loss at 2 years |  |  |  |  |
| %EBMIL^3^, mean ± SD | 77.7 ± 26.0 | 74.0 ± 24.6 | MeD=3.42 (0.50 – 6.34) | 0.022 |
| %TWL^3^, mean ± SD | 28.7 ± 9.3 | 28.3 ± 9.0 | MeD=0.21 (-0.85 – 1.28) | 0.696 |
| BMI-loss^3^, mean ± SD | 11.7 ± 4.4 | 11.9 ± 4.5 | MeD=-0.25 (-0.78 – 0.29) | 0.363 |
| Numbers are n (%) unless otherwise stated  ACS = Acute Coronary Syndrome; EBMIL = Excess BMI-loss; TWL = Total Weight Loss  1 Linear quantile mixed-effects model, adjusted for smoking; MdD, median difference.  2 Conditional logistic regression model, adjusted for smoking; OR, odds ratio.  3 Linear mixed-effects model, adjusted for smoking; MeD, mean difference. | | | |  |

* Corrected value for multiple calculations using the Bonferroni-Holm method

| **Table S4 Surgical outcomes for patients with heart failure** | | | |  |
| --- | --- | --- | --- | --- |
|  | Heart failure | Control group | *Effect size (95% CI)* | *P* |
| Number of individuals | 592 | 2960 |  |  |
| Length of hospital stay (IQR)^1^ | 1 (1-2) | 1 (1-2) | MdD=0.11 (-2.91 – 3.14) | 0.941 |
| Intraoperative complication^2^ | 23 (3.9%) | 84 (2.8%) | OR=1.41 (0.88 – 2.28) | 0.155 |
| Postoperative complication^2^ | 54 (9.3%) | 235 (8.2%) | OR=1.16 (0.85 – 1.59) | 0.342 |
| Leak/deep intraabdominal infection^2^ | 14 (2.4%) | 53 (1.8%) | OR=1.30 (0.72 – 2.36) | 0.390 |
| Bleeding^2^ | 13 (2.2%) | 63 (2.2%) | OR=0.99 (0.53 – 1.85) | 0.979 |
| Wound complications^2^ | 10 (1.7%) | 32 (1.1%) | OR=1.51 (0.72 – 3.15) | 0.278 |
| Bowel obstruction/stricture/ileus^2^ | 6 (1.0%) | 25 (0.9%) | OR=1.06 (0.42 – 2.64) | 0.904 |
| Marginal ulcer^2^ | 4 (0.7%) | 16 (0.6%) | OR=1.37 (0.44 – 4.22) | 0.586 |
| Cardiovascular complication^2^ | 7 (1.2%) | 7 (0.2%) | OR=7.90 (2.20 – 28.37) | 0.024* |
| Pulmonary complication^2^ | 6 (1.0%) | 19 (0.7%) | OR=2.10 (0.77 – 572) | 0.145 |
| Urinary tract infection^2^ | 5 (0.9%) | 10 (0.3%) | OR=2.53 (0.81 – 7.93) | 0.112 |
| Venous thrombosis^2^ | 0 (0.0%) | 6 (0.2%) | NA |  |
| Pain^2^ | 4 (0.7%) | 25 (0.9%) | OR=0.67 (0.21 – 2.13) | 0.503 |
| Malnutrition/dehydration^2^ | 4 (0.7%) | 13 (0.4%) | OR=1.68 (0.51 – 5.53) | 0.389 |
| Other complication^2^ | 2 (0.3%) | 22 (0.8%) | OR=0.50 (0.11 -2.21) | 0.359 |
| Serious postoperative complication^2^ | 24 (4.1%) | 89 (3.1%) | OR=1.19 (0.71 – 2.00) | 0.504 |
| Postoperative weight-loss at 1 year |  |  |  |  |
| %EBMIL^3^, mean ± SD | 71.3 ± 22.0 | 73.4 ± 22.4 | MeD=-1.87 (-3.93 – 0.19) | 0.076 |
| %TWL^3^, mean ± SD | 28.7 ± 8.1 | 28.3 ± 7.9 | MeD= 0.48 (-0.23 – 1.20) | 0.189 |
| BMI-loss^3^, mean ± SD | 12.5 ± 4.2 | 12.0 ± 4.0 | MeD= 0.56 (0.18 – 0.93) | 0.003 |
| Postoperative weight-loss at 2 years |  |  |  |  |
| %EBMIL^3^, mean ± SD | 71.1 ± 23.2 | 73.8 ± 24.1 | MeD=-2.52 (-5.27 – 0.22) | 0.072 |
| %TWL^3^, mean ± SD | 28.7 ± 8.9 | 28.4 ± 9.0 | MeD=0.40 (-0.62 – 1.41) | 0.446 |
| BMI-loss^3^, mean ± SD | 12.5 ± 4.5 | 12.0 ± 4.6 | MeD= 0.52 (-0.02 – 1.04) | 0.050 |
| Numbers are n (%) unless otherwise stated  EBMIL = Excess BMI-loss; TWL = Total Weight Loss  1 Linear quantile mixed-effects model, adjusted for smoking; MdD, median difference.  2 Conditional logistic regression model, adjusted for smoking; OR, odds ratio.  3 Linear mixed-effects model, adjusted for smoking; MeD, mean difference.  * Corrected value for multiple calculations using the Bonferroni-Holm method | | | |  |

| **Table S5 Surgical outcomes for patients with BMI <50** | | | |  |
| --- | --- | --- | --- | --- |
|  | Heart disease group | Control group | *Odds ratio (95% CI)* | *P* |
| Number of individuals | 1064 | 5332 |  |  |
| Length of hospital stay, median (IQR)^1^ | 1 (1-2) | 1 (1-2) | MdD=0.05 (-1.57 – 1.66) | 0.955 |
| Intraoperative complication^2^ | 31 (2.9%) | 169 (3.2%) | OR=0.90 (0.61-1.33) | 0.600 |
| Postoperative complication^2^ | 98 (9.5%) | 421 (8.1%) | OR=1.15 (0.91 – 1.45) | 0.249 |
| Leak/deep intraabdominal infection^2^ | 21 (2.0%) | 96 (1.8%) | OR=1.11 (0.68 – 1.82) | 0.665 |
| Bleeding^2^ | 28 (2.7%) | 108 (2.1%) | OR=1.26 (0.82 – 1.96) | 0.293 |
| Wound complications^2^ | 16 (1.5%) | 60 (1.2%) | OR=1.28 (0.72 – 2.27) | 0.394 |
| Bowel obstruction/stricture/ileus^2^ | 15 (1.5%) | 54 (1.0%) | OR=1.40 (0.77 – 2.53) | 0.267 |
| Marginal ulcer^2^ | 4 (0.4%) | 24 (0.5%) | OR=0.93 (0.31 – 2.78) | 0.902 |
| Cardiovascular complication^2^ | 10 (1.0%) | 10 (0.2%) | OR=6.41 (2.34 – 17.53) | <0.001* |
| Pulmonary complication^2^ | 9 (0.9%) | 33 (0.6%) | OR=1.42 (0.67 – 3.04) | 0.361 |
| Urinary tract infection^2^ | 8 (0.8%) | 15 (0.3%) | OR=2.49 (1.01 – 6.17) | 0.528* |
| Venous thrombosis^2^ | 1 (0.1%) | 7 (0.1%) | NA |  |
| Pain^2^ | 6 (0.6%) | 35 (0.7%) | OR=0.77 (0.30 – 1.94) | 0.580 |
| Malnutrition/dehydration^2^ | 7 (0.7%) | 25 (0.5%) | OR=1.39 (0.56 – 3.44) | 0.482 |
| Other complication^2^ | 9 (0.9%) | 52 (1.0%) | OR=0.84 (0.41 – 1.71) | 0.622 |
| Serious postoperative complication^2^ | 42 (4.1%) | 166 (3.2%) | OR=1.21 (0.82 – 1.80) | 0.335 |
| Postoperative weight-loss at 1 year |  |  |  |  |
| %EBMIL^3^, mean ± SD | 76.1 ± 23.6 | 74.9 ± 22.4 | MeD=1.11 (-0.44 – 2.67) | 0.161 |
| %TWL^3^, mean ± SD | 28.5 ± 8.0 | 28.0 ± 7.7 | MeD=-0.49 (-0.03 – 1.02) | 0.066 |
| BMI-loss^3^, mean ± SD | 11.7 ± 3.7 | 11.5 ± 3.5 | MeD=-0.22 (-0.02 – 0.46) | 0.077 |
| Postoperative weight-loss at 2 years |  |  |  |  |
| %EBMIL^3^, mean ± SD | 75.6 ± 25.0 | 74.9 ± 24.4 | MeD=0.54 (-1.53 – 2.62) | 0.610 |
| %TWL^3^, mean ± SD | 28.3 ± 9.0 | 28.0 ± 8.7 | MeD=-0.35 (-0.38 – 1.10) | 0.345 |
| BMI-loss^3^, mean ± SD | 11.6 ± 4.1 | 11.5 ± 4.0 | MeD=-0.18 (-0.16 – 0.52) | 0.291 |
| Numbers are n (%) unless otherwise stated  ACS = Acute Coronary Syndrome; EBMIL = Excess BMI-loss; TWL = Total Weight Loss  1 Linear quantile mixed-effects model, adjusted for smoking; MdD, median difference.  2 Conditional logistic regression model, adjusted for smoking; OR, odds ratio.  3 Linear mixed-effects model, adjusted for smoking; MeD, mean difference.  * Corrected value for multiple calculations using the Bonferroni-Holm method | | | |  |

| **Table S6 Surgical outcomes for patients with BMI >=50** | | | |  |
| --- | --- | --- | --- | --- |
|  | Heart disease group | Control group | *Odds ratio (95% CI)* | *P* |
| Number of individuals | 101 | 493 |  |  |
| Length of hospital stay, median (IQR)^1^ | 1 (1-2) | 1 (1-2) | MdD=0.16 (-0.36 – 0.67) | 0.559 |
| Intraoperative complication^2^ | 2 (2.0%) | 17 (3.4%) | OR=0.53 (0.12-2.36) | 0.410 |
| Postoperative complication^3^ | 8 (8.2%) | 43 (9.0%) | OR=0.91 (0.41-2.00) | 0.206 |
| Leak/deep intraabdominal infection^3^ | 2 (2.1%) | 8 (1.7%) | OR=1.24 (0.26-5.92) | 0.790 |
| Bleeding^3^ | 1 (1.0%) | 10 (2.1%) | OR=0.49 (0.06-3.85) | 0.496 |
| Wound complications^3^ | 1 (1.0%) | 11 (2.3%) | OR=0.44 (0.06-3.47) | 0.437 |
| Bowel obstruction/stricture/ileus^3^ | 1 (1.0%) | 4 (0.8%) | OR=1.23 (0.136-11.17) | 0.851 |
| Marginal ulcer^3^ | 0 (0.0%) | 0 (0.0%) | NA | NA |
| Cardiovascular complication^3^ | 2 (2.1%) | 2 (0.4%) | OR=5.01 (0.70-36.01) | 0.109 |
| Pulmonary complication^3^ | 2 (2.1%) | 2 (0.4%) | OR=5.01 (0.70-36.01) | 0.109 |
| Urinary tract infection^3^ | 2 (2.1%) | 4 (0.8%) | OR=2.50 (0.45-13.82) | 0.295 |
| Venous thrombosis^3^ | 0 (0.0%) | 1 (0.2%) | NA |  |
| Pain^3^ | 1 (1.0%) | 6 (1.3%) | OR=0.82 (0.10-6.88) | 0.855 |
| Malnutrition/dehydration^3^ | 0 (0.0%) | 2 (0.4%) | NA | NA |
| Other complication^3^ | 0 (0.0%) | 2 (0.4%) | NA | NA |
| Serious postoperative complication^3^ | 4 (4.1%) | 13 (2.7%) | OR=1.54 (0.49-4.82) | 0.460 |
| Postoperative weight-loss at 1 year |  |  |  |  |
| %EBMIL^4^, mean ± SD | 57.5 ± 15.3 | 58.6 ± 16.4 | MeD=-1.15 (-4.97 – 2.66) | 0.554 |
| %TWL^4^, mean ± SD | 30.7 ± 8.4 | 31.3 ± 8.7 | MeD=--0.57 (-2.61 – 1.46) | 0.582 |
| BMI-loss^4^, mean ± SD | 16.6 ± 5.2 | 17.0 ± 5.0 | MeD=-0.30 (-1.48 – 0.89) | 0.620 |
| Postoperative weight-loss at 2 years |  |  |  |  |
| %EBMIL^4^, mean ± SD | 61.1 ± 19.0 | 62.1 ± 19.9 | MeD=-1.08 (-6.74 – 4.57) | 0.707 |
| %TWL^4^, mean ± SD | 32.2 ± 9.8 | 33.1 ± 10.7 | MeD=-0.96 (-3.99 – 2.07) | 0.536 |
| BMI-loss^4^, mean ± SD | 17.1 ± 5.2 | 17.9 ± 6.2 | MeD=-0.87 (-2.61 – 0.87) | 0.325 |
| Numbers are n (%) unless otherwise stated  EBMIL = Excess BMI-loss; TWL = Total Weight Loss  1 Linear quantile mixed-effects model, adjusted for smoking; MdD, median difference.  2 Conditional logistic regression model, adjusted for smoking; OR, odds ratio.  3 Unadjusted logistic regression model due to few events in subgroups.  4 Linear mixed-effects model, adjusted for smoking; MeD, mean difference. | | | |  |
